# A 64-year-old woman with raccoon eyes following kidney biopsy: a case report

**DOI:** 10.1186/s12882-020-01770-4

**Published:** 2020-04-17

**Authors:** Shuqin Mei, Yunyang Zhao, Lin Li, Changlin Mei, Bing Dai

**Affiliations:** 1 Kidney Institute of PLA, Department of Nephrology, Shanghai Changzheng Hospital, Naval Medical University, Shanghai, China; 2 Department of Hematology, Shanghai Changzheng Hospital, Naval Medical University, Shanghai, China

**Keywords:** Raccoon eyes, Periorbital purpura, Kidney biopsy, Immunoglobulin light chain amyloidosis

## Abstract

**Background:**

Raccoon eyes or periorbital ecchymosis is caused by blood tracking into periorbital tissues, which is mostly recognized in injuries of head and neck, basal skull fractures, convexity fractures and facial fractures. It was also reported in systematic disorders, such as multiple myeloma, amyloidosis, Kaposi’s sarcoma, migraine and neuroblastoma. However, it is unusual to see a patient showing periorbital purpura after kidney biopsy with no other ecchymosis. Here, we firstly reported this rare symptom after kidney biopsy in a patient who was finally diagnosed as immunoglobulin light chain (AL) amyloidosis.

**Case presentation:**

A 64-year old woman was admitted to our clinic with 1.5 years history of sub-nephrotic proteinuria and slowly progressive deterioration of renal function. Laboratory -investigations revealed an M-peak in the λ fraction of IgA and concentrations of serum free-light-chain (FLC) were 44.95 mg/L for κ isotype and 173 mg/L for λ isotype. Unexpectedly the patient showed periorbital purpura 24 h later after kidney biopsy with no more other ecchymosis. Renal biopsy showed massively glomerulosclerosis, interstitial fibrosis with positively Congo red staining in mesangial areas. For fluorescent staining, the kidney tissue showed strongly λ light-chain deposition. The fibrils (8-12 nm in diameter) were confirmed by electron micrograph.

**Conclusions:**

This case firstly reported this rare symptom after the kidney biopsy in a patient who was finally diagnosed as AL amyloidosis. And this unique sign of periorbital ecchymosis warrants more attention as an early cue of amyloidosis.

## Background

Raccoon eyes or periorbital ecchymosis is caused by blood tracking into periorbital tissues, which is easily recognized as a common symptom of injuries of head and neck, basal skull fractures, convexity fractures and facial fractures. Also, some studies showed periorbital ecchymosis as a symptom of systematic disorders, such as multiple myeloma, amyloidosis, Kaposi’s sarcoma, migraine and neuroblastoma. The coagulopathy and vascular infiltration of amyloid fibrils in peri-orbital blood vessels in patients with amyloidosis can cause bilateral periorbital ecchymosis by minimal trauma, such as coughing, sneezing, rubbing or Valsalva maneuver. In 2017, Nasiri reported a case of bilateral periorbital ecchymosis following Endoscopic retrograde cholangiopancreatography (ERCP) and sphincterotomy [1].

Yet, periorbital ecchymosis was rare to see following kidney biopsy. Here we firstly reported a case of bilateral periorbital ecchymosis (raccoon eyes) in a 64-year old woman 24 h later after the kidney biopsy who was finally diagnosed as AL amyloidosis.

## Case presentations

A 64-year old woman presented to our clinic with 1.5 years history of proteinuria (the 24-h urine protein quantification was between 0.9–1.5 g). Renal function deteriorated gradually (the serum creatinine increased from 130 umol/L to 382 umol/L) with no history of hypertension (usually 95/60 mmHg), diabetes and other significant medical history.

**Fig. 1:**
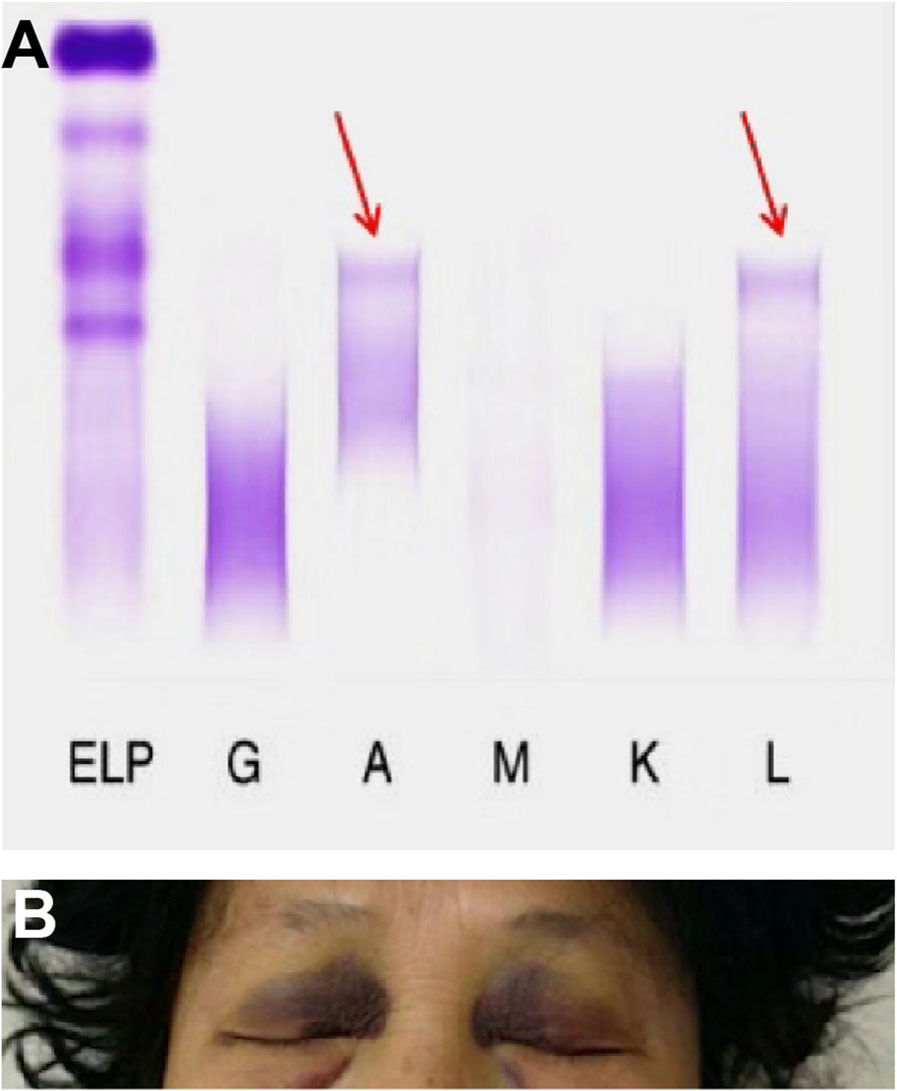
**a** M-peak in the lambda fraction of IgA with the serum lambda free light chains accumulation. **b** Photograph of the patient’s face showed bilateral periorbital ecchymosis (blue or purple discoloration of the up eyelids) following kidney biopsy

Physical examination was unremarkable. Laboratory study showed mild anemia of Hb was 97 g/L and normal coagulation test. Serum creatinine was increased at 382 umol/L and the 24-h urine protein quantification was 2.4 g. The ratio of κ/λ in the serum and urine proteins were normal. While, the testing for monoclonal protein by serum revealed an M-peak in the λ fraction of IgA (Fig. 1a). With the help of the serum free-light-chain (sFLC) assay, we got the concentrations of sFLC were 44.95 mg/L for κ isotype and 173 mg/L for λ isotype. The ratio of κ to λ was 0.26 (1.26–1.65). The bone marrow cytology test was negative. Echocardiographic findings showed the normal interventricular septum thickness which was 9 mm and EF was about 63%. Abdominal ultrasound report indicated enhanced echo of liver parenchyma and normal spleen appearance. The size of right kidney was 9.1 × 4.4 cm and the left kidney was 10 × 4.1 cm. Enhanced cortical echo, clear demarcation between cortex and medulla, normal collecting system with no pyelic separation was recorded by ultrasound. Also, electromyography and nerve conduction velocity studies showed negative results.

In order to figure out whether the kidney lesions belong monoclonal gammopathy of renal significance (MGRS), we performed kidney biopsy, and the patient showed periorbital purpura 24 h later after kidney biopsy with no more other ecchymosis (Fig. 1b).

**Fig. 2:**
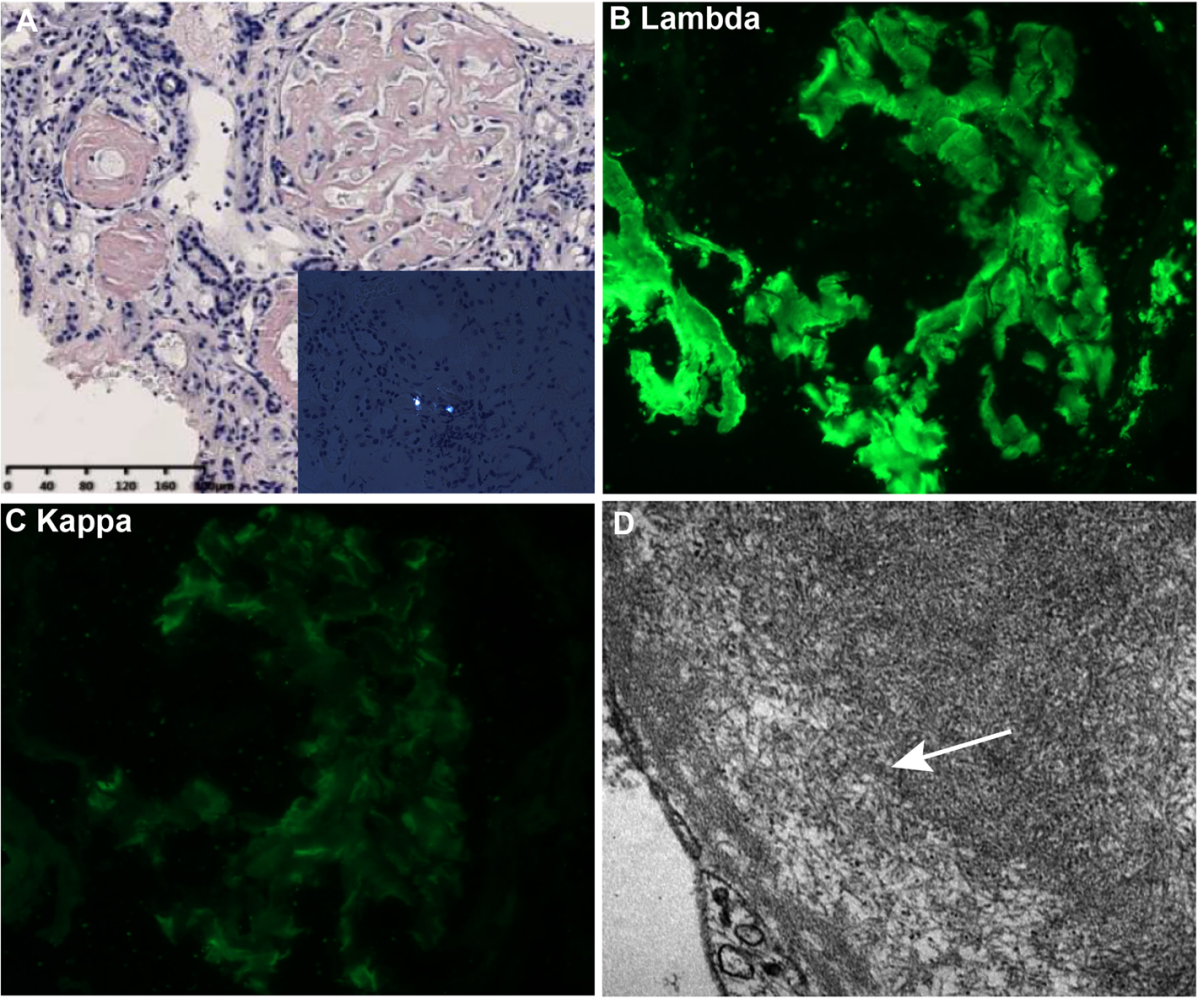
**a** Congo red staining of kidney biopsy specimen and the picture of typical apple-green birefringence under polarized microscopy (× 200). **b** and **c** Immunofluorescence microscopy showed prominent staining for lambda light chain and much weaker kappa light chain (× 400). **d** Electron micrograph showing expansion of the mesagium by amyloid fibrils, which can measure 8–12 nm in diameter. The fibrillar appearance is best appreciated at the arrow (× 10,000)

Eighteen glomeruli were sampled, all of which were glomerulosclerosis. The volume of the glomeruli was increased, accompanying with the mild to severe enlargement of mesangial areas. Renal tubular epithelial cells were swollen, degenerated and necrosis. 70–80% interstitial were fibrotic with a small number of inflammatory cells infiltration. Congo red staining was positive and typical apple-green birefringence was showed under polarized microscopy (Fig. 2a). For fluorescent staining, the kidney tissue showed strongly λ light-chain deposition (Fig. 2b). Electron micrograph showing expansion of the mesangium by amyloid fibrils, and the fibrillar appearance was best appreciated by the arrow (Fig. 2d). These fibrils (8–12 nm in diameter) are smaller than those seen in fibrillary and immunotactoid glomerulonephritis. A diagnosis of immunoglobulin light chain (AL) amyloidosis nephropathy was established. Then the patient received 2 cycles of cyclophosphamide, bortezomib, and dexamethasone. The 24-h urine protein quantification was around 0.6 g and the serum creatinine decreased to 306 umol/L.

## Discussion and conclusion

AL amyloidosis is the most common type of systemic amyloidosis, which is difficult to recognize because of the vague symptoms and range of manifestations. While, the key features of all kinds of amyloidosis is abnormal folding of a protein that is normally soluble and can be typical apple-green birefringence with polarized microscopy after Congo red dye [2, 3]. As is known to all, there are several major forms of amyloidosis, including immunoglobulin light chain (AL) amyloidosis, amyloid A (AA) amyloidosis, dialysis-related amyloidosis, heritable amyloidosis. And AL amyloidosis is the most common type of systemic amyloidosis. The pathogenesis of AL amyloidosis is an Ig light chain or the fragment of a light chain which is produced by a population of abnormal plasma cells in the bone marrow. Comparing to multiple myeloma, the cell burden in AL amyloidosis is lower, usually less than 10%. The accumulated fibrils in AL amyloidosis derive from the variable region of λ light chains in approximately 75% of cases, and κ light chains are responsible for the remainder.

Renal involvement accounts for almost 70% of patients with AL amyloidosis and most often presents as clinically apparent nephrotic syndrome and enlarged kidney size [4]. Sometimes it is insidious, only presented with proteinuria and slowly progressive deterioration of renal function with unknown etiology. So it is necessary to perform tissue biopsy (kidney, abdominal fat pad and other affected sites), once the patients with unknown renal failure accompany with monoclonal M protein or suspicious tissue infiltration.

Periorbital ecchymosis is caused by blood tracking into periorbital tissues, which are easily recognized and generally thought as a common symptom of basal skull fractures. However, it may be a sign of health threatening situations such as multiple myeloma, amyloidosis, Kaposi’s sarcoma and neuroblastoma. Usually, periorbital ecchymosis in AL amyloidosis is called “raccoon eyes” and is caused by vascular infiltration of amyloid fibrils in peri-orbital blood vessels. Other causes of peri-orbital bleeding including clotting disorders, trauma, essential thrombocytaemia [5]. Here we firstly reported this rare symptom after the kidney biopsy in a patient who was finally diagnosed as AL amyloidosis. During the kidney biopsy, the patient was asked to hold breath after inhalation, which mimicked Valsalva maneuver, and thus contribute to the periorbital purpura. Therefore, periorbital ecchymosis warrants more attention as an early cue of amyloidosis and it also can occur after an invasive diagnostic procedure such as renal biopsy.

## Data Availability

All data collected from this patient were obtained from Changzheng Hospital and are available in this paper.

## Abbreviations

AL amyloidosis: Immunoglobulin light chain amyloidosis
AA amyloidosis: Amyloid A amyloidosis
sFLC: Serum free-light-chain
ERCP: Endoscopic retrograde cholangiopancreatography
MGRS: Monoclonal gammopathy of renal significance

## References

[1] Heegaard NH (2009). Beta(2)-microglobulin: from physiology to amyloidosis. Amyloid.

[2] Nasiri J, Zamani F (2017). Periorbital ecchymosis (raccoon eye) and orbital hematoma following endoscopic retrograde Cholangiopancreatography. Case Rep Gastroenterol.

[3] Eisenberg D, Jucker M (2012). The amyloid state of proteins in human diseases. Cell..

[4] Said SM, Sethi S, Valeri AM (2013). Renal amyloidosis: origin and clinicopathologic correlations of 474 recent cases. Clin J Am Soc Nephrol.

[5] Eder L, Bitterman H (2007). Image in clinical medicine. Amyloid purpura. The New England J Med.

